# Evaluating the Highway Tunnel Construction in Western Sichuan Plateau Considering Vocational Health and Environment

**DOI:** 10.3390/ijerph16234671

**Published:** 2019-11-23

**Authors:** Peng Wu, Feng Yang, Jinlong Zheng, Yanqing Wei

**Affiliations:** 1 Department of Management Science and System Science, Sichuan University, 29 Wangjiang Road, Chengdu 610064, China; pengwu@scu.edu.cn; 2 Sichuan Highway Planning, Survey, Design and Research Institute Ltd., 1 Wuhouci Hengjie Street, Chengdu 610041, China; yf718866932@126.com (J.Z.); wayaqa@126.com (Y.W.)

**Keywords:** high altitude, environmental adaptive, vocational health, highway tunnel construction

## Abstract

Oxygen deficiency and coldness are the main challenges for highway tunnel construction in high-altitude areas such as western Sichuan plateau. The artificial oxygen supply and anti-freezing structure in the tunnel construction process has a significant impact on vocational health and the environment. Thus, the conditions of tunnels need to be carefully evaluated before construction. However, the current design code for tunnel construction contains few instructions about these aspects. This paper attempts to establish a simple evaluation method to guide the construction design by analyzing the oxygen partial pressure of trachea, the mean temperature of the coldest month, and the maximum freezing depth for tunnel projects in western Sichuan plateau. Based on the on-site meteorological monitoring at different altitudes of three typical tunnels in the western Sichuan plateau and the comparative analysis of the existing meteorological data, the corresponding relationships between the three parameters and the altitude were investigated. The thresholds by altitude for grading the tunnels are identified as 2100 m and 4200 m, respectively. The highway tunnels in the western Sichuan plateau are graded in three categories, namely, general-altitude tunnels, high-altitude tunnels, and ultra-high-altitude tunnels. The corresponding measures of oxygen supply and freezing prevention for different graded tunnels are recommended. The results would provide a basis for the design and construction of new tunnels and enhance the service life and operations safety of the tunnels in western Sichuan plateau and other similar high-altitude areas.

## 1. Introduction

Tunnels are very important for transportation in mountain areas as they can significantly reduce transportation time and enhance transportation safety. Especially in high-altitude areas where transportation is prohibited by ice and snow in winter, tunnels may make road transportation possible. However, tunnel construction in high-altitude mountain areas may cause severe vocational health problems for the workers [1] and damage to the vulnerable ecological environment [2], which make the construction extremely difficult and expensive. The main challenges for tunnel construction in this area are as follows. Worker’s health and efficiency are significantly influenced by low oxygen concentration and low temperature; machines and equipment also operate at low efficiency; extra designs such as deep-buried ditches are required to prevent damage of freezing [3,4,5]. As a result, there are not many such projects in construction. At present, no design code for high-altitude tunnel construction has been widely adopted. On the other hand, the economic development in western Sichuan plateau now generates a strong demand for transportation infrastructure. As more high-altitude highway tunnels are in design and construction in this area, there is an urgent need to study the different construction conditions for tunnels in such areas to increase construction efficiency, protect vocational health, and reduce environmental damage.

To cope with these challenges, various solutions such as artificial oxygen supply and extra heating are proposed. These solutions incur significant costs and thus should be implemented after careful consideration of local conditions and engineering requirements. The altitude of highway tunnels in western Sichuan plateau ranges from 500 m to 4400 m above sea level, and the construction conditions may vary significantly. We attempt to establish a simple grading rule for the tunnels to guide the construction design. The grading is based on analyzing oxygen deficiency and cold climate, which are key factors in high-altitude tunnels, through on-site meteorological monitoring. Since these two factors are closely related to altitude, we will use altitude as the grading criteria. Such a grading rule will be easy to implement, balance costs and efficiency, and guarantee project quality.

There already exist several grading standards for oxygen deficiency by altitude. For example, physiologists usually define altitude between 8000 and 12,000 feet (2438 m–3658 m) as high altitude, define altitude between 12,000 and 18,000 feet (3658 m–5487 m) as very high altitude, and define altitude above 18,000 feet (5487 m+) as extremely high altitude, according to Cleveland Clinic [6]. Yang et al. [7] divide altitudes into five grades according to human habitation sustainability, in which over 3600 m is the top grade, at which people are likely to have high-altitude problems. These grading standards are usually established in a laboratory environment. Nevertheless, high-altitude tunnel construction requires a heavy workload. For example, tunnel boring machine (TBM) that is widely used in city tunnel construction cannot be deployed for constructing high-altitude tunnels because it would very often get jammed by mountain rocks. Workers need to dig with equipment in hand. The work is usually done in a natural environment, where microclimate often changes due to different elevation, slope, solar radiation, cloud cover, and soil type [8]. Thus, the existing altitude grading standards may not be suitable in tunnel construction. Yang et al. [9] and Yuan [10] measure oxygen content at a particular high-altitude tunnel construction site and provide suggestions for artificial oxygen supply. But their results are collected from a single tunnel. The generality remains to be verified. It is still not clear how to overcome oxygen deficiency for tunnel construction at different altitudes.

The existing design code also has grading standards for cold climate, but the grading is not in altitude. For example, the manual for highway tunnel design in China grades regions by mean temperature in the coldest month and maximum freezing depth [11]. There are similar gradings in design codes for the railway tunnel and civil building [12,13]. These grading standards are usually established in high-latitude areas and are applied to grade a region based on meteorological data from its town area. However, western Sichuan plateau locates in low-latitude area, and the climate may change significantly even within a small region because of dramatically changing altitude and other terrain factors [14]. The meteorological data outside town area are usually unavailable. If the meteorological data in town area is used instead to guide the construction of tunnels in mountains, the errors in meteorological information may cause damage in tunnel structure [15] or extra construction cost [16]. It is, in fact, not the region but every tunnel that needs to be graded in western Sichuan plateau. However, precise information on temperature and freezing depth at the tunnel location requires meteorological monitoring for a long period, which may delay the construction period [17,18]. The altitude becomes a promising quick grading criterion for cold climate at the tunnel location as temperatures and maximum freezing depths are closely related to it. Until now, the relationship between the altitude and important indicators such as the temperature and the maximum freezing depth has not been verified in western Sichuan plateau.

In this paper, we verify the changing patterns of oxygen deficiency and cold climate as altitude ascends on the basis of meteorological monitoring near the portals of three representative tunnels in western Sichuan plateau. We use atmospheric pressure to indirectly measure oxygen deficiency. The oxygen concentration of the air remains constant at different altitudes, which is about 20.9% of the total of the air (mainly nitrogen and oxygen). But atmospheric pressure decreases as altitude ascends. As a result, the partial pressure of oxygen and the absolute amount of oxygen decreases when the atmospheric pressure decreases. The oxygen deficiency caused by low atmospheric pressure is the main reason for mountain sickness, and at extreme conditions, may cause fatal problems [19,20]. The change in oxygen concentration by altitude fits the theoretical model very well. To ensure tunnel construction efficiency and safety, we quantitatively evaluate hypoxia risks at different altitudes and propose grading rules based on our data, literature, and existing regulations. On the other hand, the changes in the temperature and freezing depth have considerable variations. After combining the data analysis and real practice of tunnel construction, it is appropriate to classify the tunnels in western Sichuan plateau into three grades, that is, general-altitude tunnel, high-altitude tunnel, and ultra-high-altitude tunnel. The thresholds for altitude grading are identified as 2100 m and 4200 m, respectively. The thresholds are relatively conservative and the corresponding construction measures recommended can protect vocational health and balance efficiency and cost.

Western Sichuan plateau plays an important role in China’s tunnel construction. Western Sichuan plateau is a plateau area located at about 28°–34° north latitude, 97°–104° east longitude. The average altitude of western Sichuan plateau is above 3000 m. Its area is about 300,000 square kilometers. Currently, more than 50% of China’s tunnels with altitude above 2500 m are in western Sichuan plateau [21,22,23]. As No. 317 and No. 318 national highways expand, which are the two main highways in the Western Sichuan Plateau, the number and length of high-altitude tunnels keep increasing in this area. Our study focuses on western Sichuan plateau but may provide insights for tunnel construction in other similar high-altitude areas.

The rest of this paper is organized as follows. Section 2 describes the methods and data. Section 3 analyzes the grading of oxygen deficiency for highway tunnel construction in western Sichuan plateau. Section 4 analyzes the grading of cold climate for highway tunnel construction in western Sichuan plateau. Section 5 summarizes the grading standard and corresponding design codes for tunnel construction. Section 6 concludes the paper.

## 2. Methods and Data

**Figure 1:**
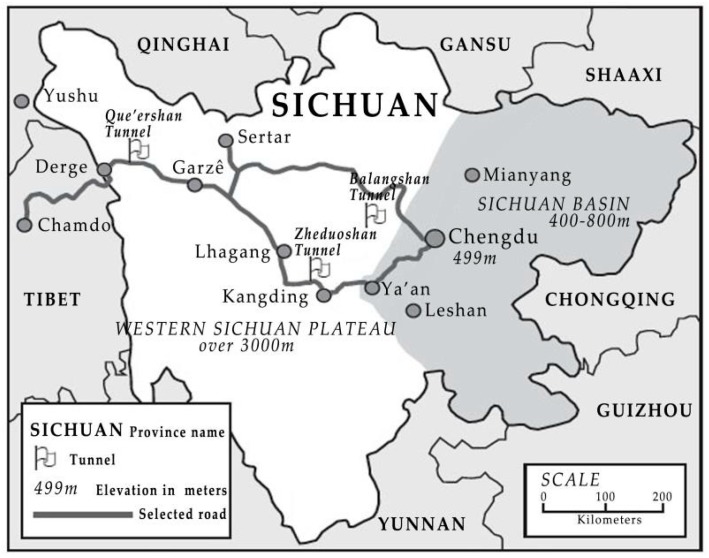
Schematic diagram of tunnel location.

We chose three long highway tunnels in western Sichuan plateau, namely Queershan Tunnel, Balangshan Tunnel, and Zheduoshan Tunnel, at which to perform on-site meteorological monitoring. The locations of these tunnels are demonstrated in Figure 1. The altitude of the three tunnels ranges from 3760 m to 4380 m.

We recorded the atmospheric pressure, temperature, and freezing depth near the three tunnels for a period of 3 to 6 years. It was a long observation for tunnel construction projects. These three variables were chosen according to the practice of tunnel construction in a high-altitude area. Atmospheric pressure determines oxygen partial pressure, which influences labor efficiency and vocational health. The monthly average temperature is required by the technical specifications for design and construction of highway in seasonally frozen soil regions from Ministry of Transport of China [24]. It is defined as the average of temperatures at 2:00, 8:00, 14:00, and 20:00 over one month in the regulation [24]. The data of temperatures at other times were used for validation of records with large deviations. Outliers were removed. These are standard procedures in meteorological observations. The monthly average temperature in the coldest month (usually in January) and the maximum freezing depth are important for anti-freezing designs and construction of tunnels. At last, the data were compared with available long-term meteorological records over the last 30 years at nearby meteorological stations. The years monitored were typical of the expected weather conditions.

**Figure 2:**
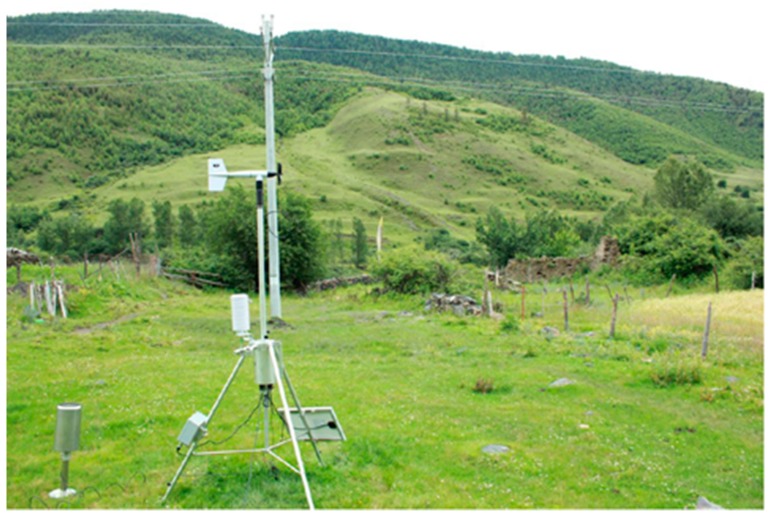
AWSTJ-3 automatic meteorological station at Zheduoshan Mountain.

The data of atmospheric pressure and temperature were captured by AWSTJ-3 automatic meteorological station using VaisalaQML sensor modules from a Finnish company. The station is demonstrated in Figure 2. The observations were made every minute during the observation periods. Its measuring range and accuracy are summarized in Table 1. The freezing depth was measured by rulers when observing the distance in the frost tube, buried vertically in the soil from the soil surface to the boundary between ice layer and unfrozen water.

The corresponding theoretic models describing the relationship between altitude and these recorded variables were then compared with our observations. Considering the microclimate at the tunnel location, the relationships between altitude and oxygen deficiency and the relationships between altitude and coldness were established and calibrated for western Sichuan plateau. After that, we proposed a grading standard for tunnels by altitude considering vocational health, labor efficiency, and costs, and recommended appropriate design codes for tunnels of different grades. At last, the applicability of the grading standard was verified by measuring the workers’ performance and the quality of tunnels.

### 2.1. Queershan Tunnel

**Figure 3:**
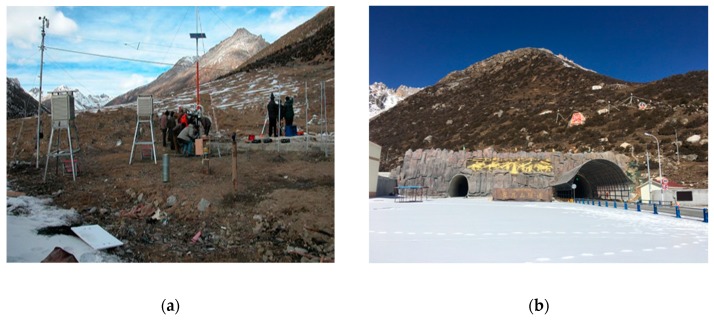
(**a**) One meteorological monitoring point in Queershan Mountain; (**b**) Portal of Queershan Tunnel.

Queershan Tunnel is located in Dege County, western Sichuan. The altitudes of its two portals are 4380 m and 4260 m, respectively. The length of this tunnel is 7079 m. The design of this tunnel began in 2002. We established monitoring points on the two sides of Queershan Mountain from 3800 m to 5200 m in altitude. A typical monitoring point is demonstrated in Figure 3a. The construction of Queershan Tunnel was completed in 2017. The portal of the tunnel is shown in Figure 3b.

We recorded the atmospheric pressure, temperature, and freezing depth at the monitoring points. The monitoring lasted for 6 years, from 2005 to 2010. The average values of meteorological monitoring results for every 100 m in altitude are reported in Table 2.

Queershan Mountain is a typical mountain in western Sichuan plateau with mountain monsoon climate. There is a clear dry season and rainy season, and local weather changes dramatically. As altitude ascends, the temperature declines at a rate of 0.55–0.65 °C/100 m. The freezing depth at the tunnel is more than 120 cm.

### 2.2. Balangshan Tunnel

**Figure 4:**
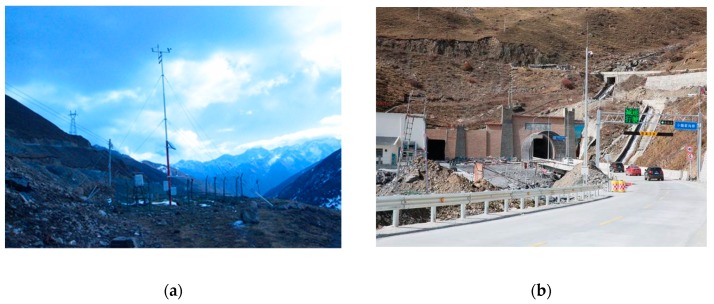
(**a**) One meteorological monitoring point in Balangshan Mountain; (**b**) Portal of Balangshan Tunnel.

Balangshan Tunnel is located in Xiaojin County, western Sichuan. The altitudes of its two portals are 3845 m and 3852 m, respectively. The length of this tunnel is 7954 m. We established monitoring points on the two sides of Balangshan Mountain from altitude 3200 m to 4200 m. A typical monitoring point is demonstrated in Figure 4a. The construction of Balangshan Tunnel was completed in 2016. The portal of the tunnel is shown in Figure 4b.

Similarly, we recorded the atmospheric pressure, temperature, and freezing depth at the observation points. The monitoring lasted for 3 years, from 2009 to 2011. The average values of meteorological monitoring results every 100 m in altitude are reported in Table 3.

Balangshan Mountain also has a clear dry season and rainy season. The mountain climate is significant with obvious vertical climate differences. The temperature decreases at a rate of about 0.6 °C/100 m as altitude increases. There are some differences between the two sides of the mountain. But the climate patterns are similar.

### 2.3. Zheduoshan Tunnel

Zheduoshan Tunnel is located in Yulin Town. The altitudes of its two portals are 3760 m and 3880 m, respectively. The length of this tunnel is 8427 m. We established monitoring points on the two sides of Zheduoshan Mountain from 3400 m to 4200 m. The construction of Zheduoshan Tunnel began in 2018. Now, it is still in the construction process.

The monitoring lasted for 3 years, from 2012 to 2015. The average atmospheric pressure, temperature, and freezing depth every 100 m in altitude are reported in Table 4.

The climate at Zheduoshan Tunnel has a similar pattern as Queershan Tunnel and Balangshan Tunnel. The temperature on the west side is slightly higher than on the east side and drops more slowly as altitude ascends. The maximum freezing depth is larger on the east side than on the west side. The differences are because of changes in microclimate near the tunnel influenced by elevation, slope, solar radiation, cloud cover, soil type, and so forth.

In summary, the three tunnels share similar mountain monsoon climate though located hundreds of kilometers away and at different altitudes. There are minor differences in atmospheric pressure, temperature, and freezing depth, but the patterns as altitude ascends are similar. These data provide a basis for our grading analysis.

## 3. Grading Oxygen Deficiency for Highway Tunnel Construction in Western Sichuan Plateau

The grading of oxygen deficiency in tunnel construction is based on the performance of labor efficiency and vocational health. As altitude ascends, oxygen deficiency will cause a decrease in labor efficiency and even severe health problems.

The oxygen partial pressure is regarded as the main indicator of oxygen deficiency. It can be calculated using atmospheric pressure. The formula of calculation is shown in Equation (1).

P_O2_ = F_O2_(P − 6.27) (1)

where P_O2_ represents the oxygen partial pressure in kPa, F_O2_ is the oxygen concentration, and P is the atmospheric pressure in kPa [25]. Consequently, the equivalent sea-level oxygen concentration at atmospheric pressure P can be calculated as

F’_O2_ = (P − 6.27) × 20.9%/(101.325 − 6.27) (2)

**Figure 5:**
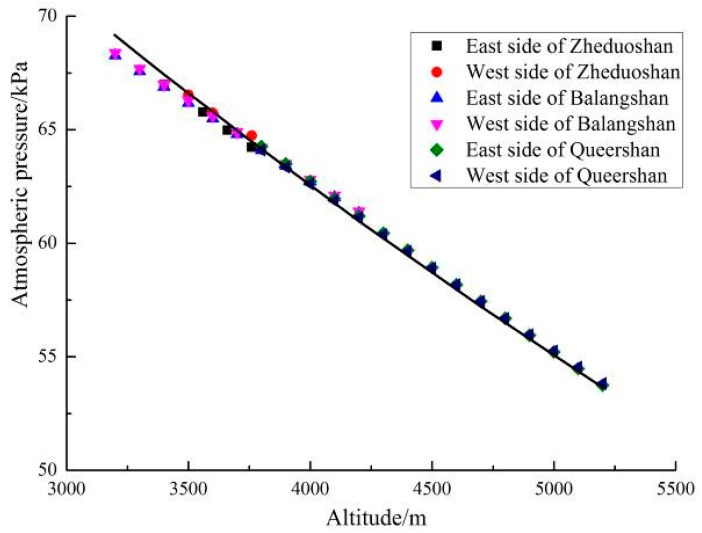
Relationship between atmospheric pressure and altitudes.

We compared the data in Table 1, Table 2 and Table 3 to the model in U.S. Standard Atmosphere 1976 [26]. The data fits the model very well, and the relationship between atmospheric pressure and altitude can be expressed in Equation (3) and Figure 5.

P = 101.325 × (1 − h/44329)^5.1^ (3)

The variable h represents the altitude in meters. The standard error of the estimated coefficient of 5.1 is 0.011, thus the coefficient is significant (*p*-value < 0.05). The adjusted R-square for this nonlinear regression is 0.99508.

Our grading standard is based on the effects of acute exposure to oxygen-deficient atmospheres on human beings [27]. The physiological effects are summarized in Table 5.

In many countries including China, the legal oxygen concentration limit for workplace exposure is 19.5% [28]. According to the physiological effects, we identify 16% and 12% as another two critical thresholds for labor efficiency and vocational health in tunnel constructions. If the equivalent sea-level oxygen concentration is less than 16%, there is slight oxygen deficiency and labor efficiency will be negatively affected. According to the experience in construction projects with oxygen deficiency, the workers’ labor time reduces from 8 h per day to about 5 h per day. If the equivalent sea-level oxygen concentration is less than 12%, then there is severe oxygen deficiency and the workers’ health will be damaged. Consequently, 12%, 16%, and 19.5% of equivalent sea-level oxygen concentration were selected as our grading thresholds.

By Equations (2) and (3), we translated the oxygen concentration into atmospheric pressure, and then into altitude. The altitude 600 m corresponds to the oxygen concentration of 19.5%. The altitude of 2100 m corresponds to the oxygen concentration of 16%. And the altitude 4200 m corresponds to the oxygen concentration of 12%. Tunnels at different altitudes will be graded at different categories. The grading of oxygen deficiency for tunnels by altitude is summarized in Table 6.

Guo et al. [29] provided a similar grading standard of oxygen deficiency for tunnel construction through theoretical analysis. By contrast, our thresholds in altitude for grading oxygen deficiency are based on field data observed at the tunnel location. Compared with their grading thresholds 2500 m and 4500 m, our thresholds 2100 m and 4200 m are more conservative. This difference is because the observed atmospheric pressure under 3500 m in western Sichuan plateau is slightly lower than the theoretical prediction, thus the oxygen partial pressure is lower. And, we prefer to be more conservative when defining the altitude threshold which threatens workers’ health.

## 4. Grading of Cold Climate for Highway Tunnel Construction in Western Sichuan Plateau

Cold climate is another main challenge for tunnel construction at high altitude. In order to adapt to the cold climate, more engineering work such as insulation or deep draining system is required, thus increases the cost and burden to the local ecological environment. The grading of the cold climate is based on the average temperature of the coldest month and the maximum freezing depth. As altitude ascends, the average temperature of the coldest month will decrease and the maximum freezing depth will increase. To control tunnel construction cost and environmental burden, we quantitatively measured coldness at different altitudes and propose grading rules based on our data, literature, and existing design codes.

**Figure 6:**
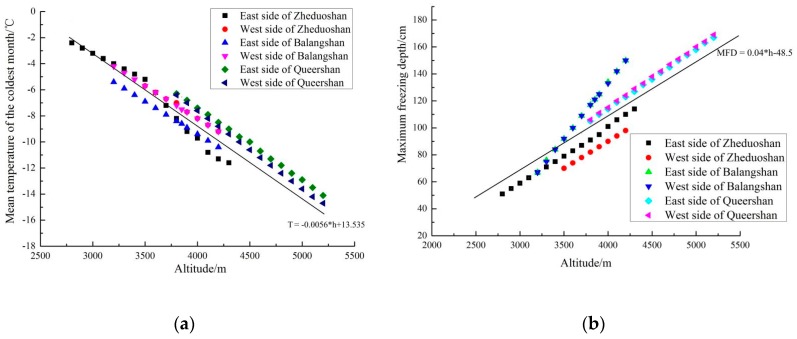
(**a**) The relationship between mean temperature of the coldest month and altitude; (**b**) The relationship between maximum freezing depth and altitude.

We ran regressions using the data in Table 2, Table 3 and Table 4 to evaluate the relationship between coldness and altitude in western Sichuan plateau. The results are demonstrated in Figure 6.

At the locations of all the tunnels, the average temperature of the coldest month decreases linearly in altitude. According to the U.S. Standard Atmosphere 1976, the temperature decreases by 0.65 °C every 100 m higher for altitudes below 11,000 m [26]. From the data of the three tunnels, we found the temperature decreasing rate is between 0.55 °C to 0.65 °C per 100 m, which is very close to the existing model predictions, but there are minor variations at different tunnels. The altitude–temperature relationship can be represented by the following equation after regression,

T = −0.0056 × h + 13.535(4)

where T represents the average temperature of the coldest month in °C, h is the altitude in meters. The standard error of the estimated slope is 2.26 × 10^−5^, thus the coefficient is significant (*p*-value < 0.05). The adjusted R-square is 0.99729. The simple linear equation fits the data very well, and the line of Equation (4) is drawn in Figure 6a.

According to Code for Thermal Design of the Civil Building in China (GB50176-2016), regions with the average temperature in the coldest month less than −10 °C are classified as severe cold area, regions with the average temperature in the coldest month between 0 °C and −10 °C are cold area [30]. This is a conservative standard compared with other design codes. We used the two threshold temperatures 0 °C and −10 °C to derive the threshold altitudes. From Equation (4), the altitudes which correspond to temperature 0 °C, −10 °C are approximately 2400 m, 4200 m, respectively.

The maximum freezing depth directly determines the effort required in the construction. For example, if the maximum freezing depth is large, the ditches should be buried deeper to avoid damages from freezing. The maximum freezing depth depends on many factors such as the local climatic conditions, the heat transfer properties of the soil and construction materials, and nearby heat sources. As far as we know, there is no simple formula describing the relationship between maximum freezing depth and altitude. As demonstrated in Figure 6b, the six groups of data monitored at the three tunnels show that the maximum freezing depth also decreases linearly in altitude. We conjecture that at least in western Sichuan plateau, this linear relationship is common at different tunnels. However, both the slope and intercept of the linear functions vary within a considerable range at different locations. It may be caused by nearby terrestrial heat or different types of soil. The relationship between altitude and maximum freezing depth is estimated by the following equation after regression,

MFD = 0.04 × h − 48.5(5)

where MFD is the maximum freezing depth in centimeters, h is the altitude in meters. The standard error of the estimated slope is 4.04 × 10^−4^, thus the coefficient is significant (*p*-value < 0.05). The adjusted R-square is 0.99487. The linear Equation (5) fits the data very well and is drawn in Figure 6b.

The grading of maximum freezing depth in existing design codes for highway and railway tunnels in China cannot be applied directly in western Sichuan plateau because those standards are mainly for high-latitude areas. The maximum freezing depth is significantly smaller in low-latitude areas such as western Sichuan plateau. We thus derived the thresholds to grade maximum freezing depth from construction practice using the relationship between altitude and maximum freezing depth.

**Figure 7:**
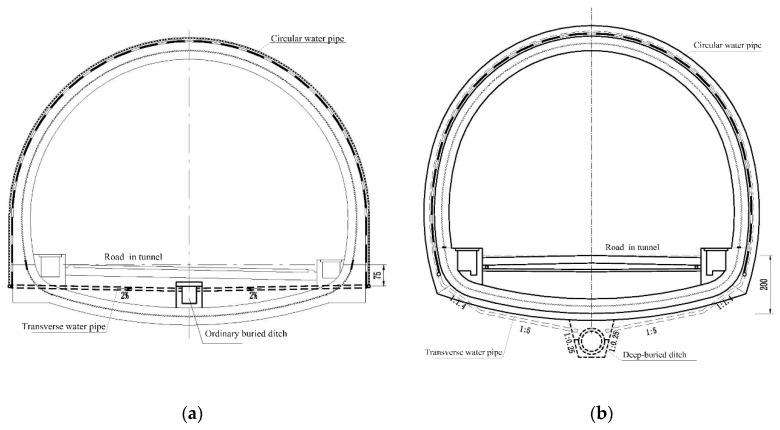
(**a**) Ordinary ditch; (**b**) Deep-buried ditch.

**Figure 8:**
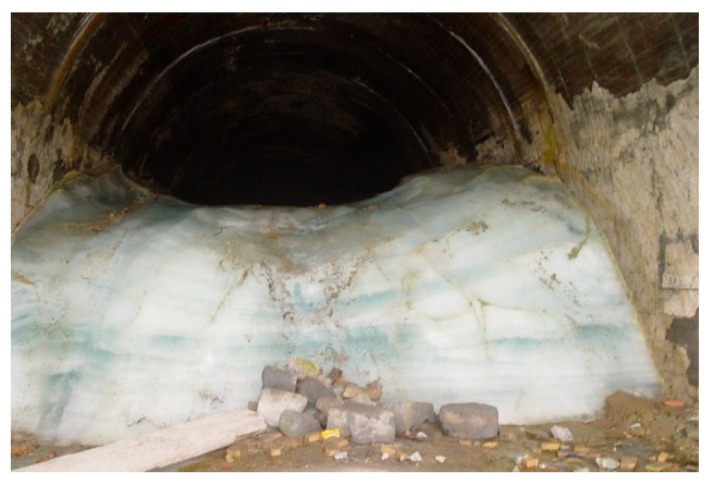
A frozen tunnel when ditches are frozen.

In the practice of tunnel construction in western Sichuan plateau, there are two kinds of ditches in the design, as shown in Figure 7. One is an ordinary ditch buried at the depth of 50 cm–65 cm, which is used in an ordinary environment. The other is deep-buried ditch at the depth of 150 cm–200 cm, which is used in a severe-cold environment. If the ditch is buried shallower than the maximum freezing depth, it may be frozen and stops functioning (see Figure 8). We used these depths to derive the corresponding threshold altitudes. To be conservative, we used the lower bound depths 50 cm and 150 cm to derive the corresponding threshold altitudes. Because the thermal conductivity of concrete is higher than soil, the freezing depths in concrete are about 1.3 times larger than the freezing depths in the soil. Hence the freezing depths 50 cm and 150 cm in concrete were transformed to freezing depths 38 cm and 115 cm in soil. We used Equation (5) to derive conservative thresholds to grade altitudes. The altitudes which correspond to maximum depths 38 cm and 115 cm are approximately 2100 m and 4300 m, respectively.

The ditch should be buried below the maximum freezing depth, otherwise, it may be damaged after freezing. According to our grading using maximum freezing depth data, it is safe to use ordinary ditch for tunnels below 2100 m in altitude and to use deep-buried ditch for tunnels between 2100 m and 4300 m in altitude.

To guarantee the service life and operations safety of the tunnel, we took the minimum of the thresholds derived from the two variables of coldness as a conservative standard. Tunnels below 2100 m were categorized as in an ordinary environment. Tunnels between 2100 m and 4200 m were categorized as in a cold environment. Tunnels above 4200 m were categorized as in a severely cold environment.

## 5. Recommendations for Graded Tunnel Construction in Western Sichuan Plateau

Through grading the oxygen deficiency level and coldness, we concluded that the altitudes 2100 m and 4200 m are important thresholds which may be used to guide different construction measures. We classified tunnels lower than 2100 m as ordinary-altitude tunnels, defined tunnels between 2100 m and 4200 m as high-altitude tunnels, and defined tunnels above 4200 m as ultra-high-altitude tunnels. The results are summarized in Table 7.

We verified the grading standard by further observations in the tunnels. According to Table 7, Queershan Tunnel is graded as an ultra-high-altitude tunnel and Balangshan Tunnel and Zheduoshan Tunnel are graded as high-altitude tunnels. Three workers were enrolled in the verification process as subjects. Their physiological performance was measured when they were working at the bottom of Zheduoshan mountain, at Balangshan Tunnel, and at Queershan Tunnel. The heart rate and arterial pressure were measured by Omron HEM-7201 blood pressure monitor. The accuracy of the heart rate is ±5%, and the precision of arterial pressure is ±3 mmHg of reading. The saturation of blood oxygen was measured by Likang POD2 blood oxygen saturation monitor. The accuracy of SaO2 is ±2% of reading. Each worker was measured three times at one location. The results are summarized in Table 8.

Table 8 shows that at high-altitude tunnels, the saturation of blood oxygen drops by about 12%, the heart rate and the arterial pressure increase by about 12% compared with the data at sea-level locations. It was observed that the workers needed to rest more frequently and the labor efficiency was significantly lower. At ultra-high-altitude tunnels, the saturation of blood oxygen drops by about 20%, the heart rate and the arterial pressure increase by about 18% and 17%. According to Shen et al.’s study on workers’ performance in the construction of plateau railway, when SaO2% drops to 75%–80%, the muscle functioning is influenced and there are lethal risks [31]. We observed that in the ultra-high-altitude tunnel, the workers could not continue working without support in oxygen supply. This was not a formal physiological study. Only three workers took part in the measurement at the three locations. Since the subjects were limited, we were not able to establish any statistical robustness from the data. It just provides several representative cases/examples of workers at different graded tunnels, which partially support the grading threshold we proposed.

The grading of tunnels is also safe for the cold climate in western Sichuan plateau. According to the operations records of the existing tunnels in western Sichuan plateau, the anti-freezing measures for different grades of tunnels function very well. No significant damage to the tunnel itself and the ecological environment has been observed.

We make the following suggestions for tunnel construction work, combining the altitude grading standard and our work experience on existing tunnels.

The ordinary tunnel construction does not require extra oxygen supply or anti-freezing measures. The level of oxygen deficiency is slight that it does not affect labor efficiency. The level of coldness does not affect the normal functioning of the draining system.

The high-altitude tunnel construction should be supported by oxygen supply for workers and anti-freezing measures. We recommend using diffusive oxygen supply in partial places of the tunnel to maintain labor efficiency and using insulation for ditches and portals to prevent freezing problems. Such measures can effectively support labor efficiency [32] and prevent water leakage outside the tunnel, thus reduce the impact on the local ecological environment.

The ultra-high-altitude tunnel construction requires a comprehensive oxygen supply mechanism, which consists of partial diffusive oxygen supply and personal mask oxygen supply. Meanwhile, the ultra-high-altitude tunnel also requires integrated anti-freezing and frost resistance measures such as insulation, deeply buried ditch, surrounding rock grouting, and antifreeze lining. With these measures, the labor efficiency can be maintained at a much lower but acceptable level. As a result, the duration of construction will be extended by approximately 20%. The costs for vocational health protection and anti-freezing measures also increase significantly for ultra-high-altitude tunnels, by about 20% compared with similar tunnels at the sea level. There will be great challenges for design and construction, thus we suggest avoiding tunnel construction in altitudes above 4200 m.

The suggestions and their achieved improvements are summarized in Table 9.

## 6. Conclusions

To cope with challenges from oxygen deficiency and coldness in highway tunnel construction in western Sichuan plateau, we provide a simple grading rule of tunnels by altitude to guide the construction process. The grading rule is derived by analyzing the oxygen partial pressure of trachea, the mean temperature of the coldest month, and the maximum freezing depth for tunnel projects after on-site meteorological monitoring. These three variables exhibit a good linear relationship with altitudes, thus can be graded through one altitude variable.

The critical threshold altitudes for tunnel grading, 2100 m and 4200 m, are consistent with existing design codes but can be used to provide more guidance for oxygen supply and anti-freezing engineering in high-altitude tunnel construction. Different means are recommended for ordinary tunnels, high-altitude tunnels, and ultra-high-altitude tunnels, respectively. The results may help to accelerate new tunnel construction at high altitudes by avoiding long-term meteorological monitoring while controlling vocational health risks and project quality. The grading standard of tunnels by altitude can be easily applied in future tunnel construction in western Sichuan plateau area and other areas with a similar environment.

## Figures and Tables

**Table 1:** Measuring range and accuracy of AWSTJ-3 station.

Variable	Measuring Range	Resolution	Error
atmospheric pressure	450 hPa–1060 hPa	0.1 hPa	±0.3 hPa
temperature	−50 °C–+60 °C	0.1 °C	±0.2 °C

**Table 2:** Meteorological monitoring results of Queershan tunnel.

Altitude	Atmospheric Pressure (kPa)		Mean Temperature in the Coldest Month (°C)		Maximum Freezing Depth (cm)	
(m)	East Side	West Side	East Side	West Side	East Side	West Side
3800	64.26	64.11	−6.3	−6.4	105	106
3900	63.49	63.36	−6.8	−7.0	110	111
4000	62.73	62.61	−7.4	−7.6	114	115
4100	61.96	61.87	−7.9	−8.2	119	120
4200	61.20	61.13	−8.5	−8.8	123	124
4300	60.44	60.38	−9.0	−9.4	127	129
4400	59.69	59.64	−9.6	−10.0	132	133
4500	58.93	58.90	−10.0	−10.6	136	138
4600	58.18	58.17	−10.7	−11.2	141	142
4700	57.44	57.43	−11.3	−11.8	145	147
4800	56.69	56.69	−11.8	−12.4	150	151
4900	55.95	55.97	−12.4	−13.0	154	155
5000	55.21	55.25	−12.9	−13.6	158	160
5100	54.48	54.54	−13.5	−14.2	163	164
5200	53.75	53.82	−14.1	−14.7	167	169

**Table 3:** Meteorological monitoring results of Balangshan tunnel.

Altitude	Atmospheric Pressure (kPa)		Mean Temperature in the Coldest Month (°C)		Maximum Freezing Depth (cm)	
(m)	East Side	West Side	East Side	West Side	East Side	West Side
3200	68.27	68.39	−5.4	−4.2	67	67
3300	67.57	67.69	−5.9	−4.7	76	75
3400	66.88	66.99	−6.4	−5.2	84	84
3500	66.18	66.29	−6.9	−5.7	92	92
3600	65.49	65.60	−7.4	−6.2	100	100
3700	64.80	64.90	−7.9	−6.7	109	109
3800	64.10	64.20	−8.4	−7.2	117	117
3900	63.41	63.50	−8.9	−7.7	125	125
4000	62.71	62.80	−9.4	−8.2	134	133
4100	62.02	62.10	−9.9	−8.7	142	142
4200	61.32	61.40	−10.4	−9.2	150	150

**Table 4:** Meteorological monitoring results of Zheduoshan tunnel.

Altitude	Atmospheric Pressure (kPa)		Mean Temperature in the Coldest Month (°C)		Maximum Freezing Depth (cm)	
(m)	East Side	West Side	East Side	West Side	East Side	West Side
3400	66.80	66.87	−4.8	−5.1	75	67
3500	65.90	66.20	−5.2	−5.7	79	70
3600	65.20	65.40	−6.2	−6.2	83	74
3700	64.40	64.50	−7.2	−6.7	87	78
3800	63.95	63.97	−8.2	−7.0	91	82
3900	63.57	63.67	−9.2	−7.7	95	86
4000	63.10	62.95	−9.7	−8.2	101	90
4100	62.87	62.55	−10.8	−8.7	106	94
4200	62.56	62.23	−11.3	−9.2	110	98

**Table 5:** Physiological effects of acute exposure to oxygen-deficient atmospheres.

Effect	Equivalent Sea-Level Oxygen Concentration (%)
No symptoms	16–20.9
Increased heart and breathing rate, some loss of coordination, increased breathing volume, impaired attention and thinking	16
Abnormal fatigue upon exertion, emotional upset, faulty coordination, impaired judgment	14
Very poor judgment and coordination, impaired respiration that may cause permanent heart damage, nausea, and vomiting	12
Nausea, vomiting, lethargic movements, perhaps unconsciousness, inability to perform vigorous movement or loss of all movement, unconsciousness followed by death	<10

**Table 6:** Oxygen deficiency for tunnel construction at different altitudes in western Sichuan plateau.

Altitude (m)	The Oxygen Partial Pressure of Trachea (kPa)	Equivalent Sea-Level Oxygen Concentration (%)	Level of Oxygen Deficiency
<600	>18.44	>19.5	No oxygen deficiency
600–2100	15.22–18.44	16–19.5	Slight oxygen deficiency
2100–4200	11.44–15.22	12–16	Medium oxygen deficiency
>4200	<11.44	<12	Severe oxygen deficiency

**Table 7:** Tunnel grading by altitudes in western Sichuan plateau.

Tunnel Classification	Altitude(m)	Level of Oxygen Deficiency	Level of Coldness
Ordinary tunnel	<2100	Slight oxygen deficiency	Ordinary
High-altitude tunnel	2100–4200	Medium oxygen deficiency	Cold
Ultra-high-altitude tunnel	>4200	Severe oxygen deficiency	Severe cold

**Table 8:** Physiological performance of tunnel workers in western Sichuan plateau.

Location	Altitude (m)	Equivalent Sea-Level Oxygen Concentration (%)	Saturation of Blood Oxygen (SaO2, %)	Heart Rate (BPM)	Mean Arterial Pressure (mmHg)
Sea-level	50	20.9	97.23 ± 1.36	77.6 ± 8.37	87.5 ± 12.83
Balangshan Tunnel	3850	12.5	85 ± 3.25	87 ± 7.64	98.5 ± 6.83
Queershan Tunnel	4380	11.6	77.85 ± 6.02	91.7 ± 10.07	102.0 ± 8.24

**Table 9:** Suggestions for constructing tunnels of different grades.

Tunnel Classification	Artificial Oxygen Supply	Anti-Freezing Measures	Improvements
Ordinary tunnel	Not required	Not required	-
High-altitude tunnel	diffusive oxygen supply	Insulation for ditches and portals	Maintain labor efficiency, less environmental impact
Ultra-high-altitude tunnel	comprehensive oxygen supply mechanism	insulation, deeply buried ditch, surrounding rock grouting and anti-freeze lining	Maintain labor health, resist frost
